# Comparative study of torque expression among active and passive self-ligating and conventional brackets

**DOI:** 10.1590/2177-6709.20.6.068-074.oar

**Published:** 2015

**Authors:** Érika Mendonça Fernandes Franco, Fabrício Pinelli Valarelli, João Batista Fernandes, Rodrigo Hermont Cançado, Karina Maria Salvatore de Freitas

**Affiliations:** 1 Graduate student, Faculdade Ingá (UNINGÁ), Department of Orthodontics, Maringá, Paraná, Brazil; 2 Professor, Faculdade Ingá (UNINGÁ), Department of Orthodontics, Maringá, Paraná, Brazil; 3 Mechanical Engineer, Universidade Federal de Itajubá (UNIFEI), Itajubá, Minas Gerais, Brazil

**Keywords:** Torque, Orthodontic appliance design

## Abstract

**Objective:**

The aim of this study was to compare torque expression in active and passive self-ligating and conventional brackets.

**Methods:**

A total of 300 segments of stainless steel wire 0.019 x 0.025-in and six different brands of brackets (Damon 3MX, Portia, In-Ovation R, Bioquick, Roth SLI and Roth Max) were used. Torque moments were measured at 12°, 24°, 36° and 48°, using a wire torsion device associated with a universal testing machine. The data obtained were compared by analysis of variance followed by Tukey test for multiple comparisons. Regression analysis was performed by the least-squares method to generate the mathematical equation of the optimal curve for each brand of bracket.

**Results:**

Statistically significant differences were observed in the expression of torque among all evaluated bracket brands in all evaluated torsions (*p* < 0.05). It was found that Bioquick presented the lowest torque expression in all tested torsions; in contrast, Damon 3MX bracket presented the highest torque expression up to 36° torsion.

**Conclusions:**

The connection system between wire/bracket (active, passive self-ligating or conventional with elastic ligature) seems not to interfere in the final torque expression, the latter being probably dependent on the interaction between the wire and the bracket chosen for orthodontic mechanics.

## Resumo

**Objetivo:**

o objetivo deste estudo foi comparar a expressão do torque em braquetes autoligáveis ativos e passivos e nos convencionais.

**Métodos:**

no total, 300 segmentos de fio de aço inoxidável 0,019" x 0,025" foram usados. Seis diferentes marcas de braquetes foram analisadas (Damon 3MX, Portia, In-Ovation R, Bioquick, Roth SLI, Roth Max). Os momentos de torque foram medidos em 12^0^, 24^0^, 36^0^ e 48^0^, utilizando-se um dispositivo de torção de fio associado a uma máquina de ensaios universal. Os dados obtidos foram comparados pela análise de variância, seguida de teste de Tukey para comparações múltiplas. A análise de regressão foi realizada pelo método dos mínimos quadrados, para gerar uma equação matemática da curva ótima de cada marca de braquete.

**Resultados:**

foram observadas diferenças estatisticamente significativas na expressão do torque entre todas as marcas de braquetes avaliadas e em todas as torções testadas (*p* < 0,05). Encontrou-se que o Bioquick apresentou a menor expressão do torque em todas as torções testadas; em contraste, o braquete Damon 3MX apresentou a maior expressão do torque até a torção de 36^0^.

**Conclusões:**

o sistema de ligação entre fio e braquete (autoligável ativo ou passivo, ou convencional com ligadura elástica) parece não interferir na expressão final do torque, a qual é dependente da interação entre o fio e o braquete escolhido para ser utilizado na mecânica ortodôntica.

## INTRODUCTION

Torque is a moment generated by the torsion of a rectangular wire in the bracket slot. In Orthodontics, it represents the buccolingual inclination of a tooth's root/crown and it is an orthodontic adaptation used to describe the rotation around the X-axis.^1^

Theoretically, third-order moments can be calculated from the nominal dimensions of arches and brackets. It has been shown that there is a significant discrepancy between what has been accomplished in theory and what is seen clinically between bracket/wire. These variations in torque can be attributed to many factors, such as bracket design, clearance (or play) between the wire and the slot, slot dimension, ligature mode, bracket deformation, wire stiffness, torsion magnitude and wire size.^2^^-^ 5 Other factors also have an impact on third-order moments, including bracket bonding errors^6^ and irregularities in the morphology of tooth crown.^7^^,^ 8

Some studies have found only minor differences among the various systems of brackets, active and passive self-ligating brackets as well as conventional ones, with respect to their effectiveness in relation to torque expression and correction.^9^ It was also found that self-ligating brackets are not superior in relation to conventional ones; particularly to what concerns their biomechanical characteristics, there are no statistically significant differences between them.^10^^,^ 11

Whereas some of these systems seem to have less friction *in vitro*, their torque characteristics have not yet been studied in detail.^12^ Due to the complexity of the experimental setup, there have been only small studies conducted to evaluate torque expression, and numerical analyses have not been carried out for the expression of torque in various types of self-ligating brackets.^7^^,^ 13

Thus, the objective of the present study was to compare the expression of torque in active and passive self-ligating as well as conventional brackets, generated at different torsion angles of the wire. Comparisons regarding torque expression among brackets of different brands were also performed. Furthermore, it was found the range of torsion angles corresponding to the torque ranges considered clinically effective. 

## MATERIAL AND METHODS

The sample used in this *in vitro* study comprised 30 maxillary right central incisor brackets of six distinct brands (five self-ligating and one conventional). Among the self-ligating brackets tested (5 of each brand), Damon 3MX (Standard torque, Ormco, USA) and Portia (3M, Abzil, Brazil) were considered by the manufacturers as passive; while In-Ovation R (GAC, USA), Bioquick (Forestadent, Germany) and Roth SLI (Morelli, Brazil) were considered as active. The conventional bracket tested (n = 5) was Roth Max (Morelli, Brazil), used as a control. The ligation method used in conventional brackets was the elastomer. Slot size of all brackets was 0.022 x 0.028-in. The tested brackets were purchased randomly, instead of being donated, in order to avoid potential influence from manufacturers in the final results. 

Three hundred stainless steel wire segments were used (Morelli, Sorocaba, SP, Brazil), each one 3.5 cm in length; and a rectangular section of 0.019 x 0.025-in, so that, for each test, a different segment of wire was used.

Stainless steel metal cylinders were manufactured, each one 4 cm in length and approximately 1 cm in diameter, in order to have accessories bonded to them. Grooves were made on the base surface of these cylinders for better adhesion of brackets. Brackets were bonded to the cylinders by Araldite Hobby Epoxy glue. 

The prescription of bracket torque did not affect our study, since the position of zero torque was used as a baseline to start every test. In order to ensure this, bonding was supported by a mounting device. This device consisted of a calibrator which was adapted and fitted to the walls of the bracket slot, causing it to achieve three-dimensional alignment. It provided bonding to the metallic cylinder that neutralized torque and pre-existing angles of each bracket.

A universal testing machine EMIC DL2000 (Instron Brazil scientific equipament - Paraná - Brazil) was used and adapted to it; a device for torsion tests of wires and brackets was developed to perform the torsion of the wire on both extremities symmetrically. In each test, the machine was reset in the system, and new leveling was performed in order to avoid residual forces and torques of the previous test. After this process, the test was started by adapting the cylinder with bracket to the base of the device, and inserting the wire segment being tested. The system was fixed by screws to prevent dislocations or slides in the set, which could alter the results.

**Figure 1:**
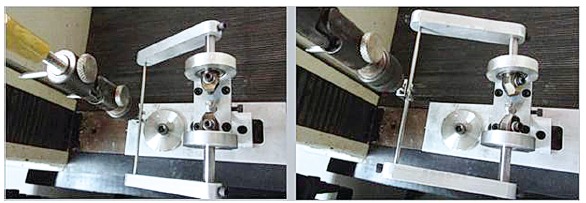
- Torsion device.

The movement (ascent) of the tailstock of the universal testing machine was transmitted to the torsion device by an articulated rod under 0.25 rpm (Fig 1). 

A transducer for measurement of force/torque, with strain gauge load cell was used to measure the components of forces and moments (torque) of the bracket/wire combinations tested. The load cell used was "Trd 19" 200 N. There was a sensor that could capture the applied force and torques, as well as the torsion angle, and electronically transfer it in the form of data to a computer, so the results were translated and displayed in graphical form. A data-capture software (version 3.01 Tesc) was used to capture the signal from the transducer and register it to the file. 

Each bracket/wire combination was tested ten times, and, for each torsion, a different wire segment was inserted. The combinations were tested in different torsions: 12°, 24°, 36° and 48°, and for every torsion angle in the wire, the moment of force was rated in Nmm.

### Statistical analysis 

Assessment of data normality was performed through Kolmogorov-Smirnov test, followed by parametric tests. Intergroup comparisons were conducted by one-way ANOVA and Tukey tests.

Regression was performed by the least-squares method in order to assess the trend and the behavior of variables of each brand tested. The coefficient of determination R^2^ was employed to assess regression quality and check if the regression found corresponded to the best representative curve of the correlation between the two variables, which explains, in percentage, the relationship between variables (R^2^ ≥ 0 ≤ 1). Regression was performed by the least-squares method to generate the mathematical equation of the optimal curve of each bracket brand separately. By the equations, both the angles of initial torque records and the angles regarding torque range that were considered clinically effective were calculated by the Tartaglia-Cardano method. 

Statistical analyses were performed with Statistica software (Statistica for Windows 6.0; Statsoft, Tulsa, Oklahoma, USA). Results were considered statistically significant for *p* < 0.05. Regression and equations were obtained in Excel 2007 software. 

## RESULTS

### Intergroup comparisons

At 12°, Bioquick obtained a smaller force *x* deformation ratio (2.90 Nmm). Shortly thereafter, Portia, with 5.07 Nmm, followed by Roth SLI, with 6.74 Nmm. Roth Max and In-Ovation R brackets came afterwards with statistically similar results (9.97 and 9.14 Nmm, respectively). Damon 3MX showed the greatest force *x* deformation relationship (12.27 Nmm) (Table 1). 

**Table 1:** - Intergroup comparison of different brackets used in the trials in the four torsions tested (one-way ANOVA test followed by Tukey test).

**Variables**	**Roth Max**	**Roth SLI**	**Damon MX**	**In-Ovation R**	**Bioquick**	**Portia**	***p*-value**
	**(n = 50)**	**(n = 50)**	**(n = 50)**	**(n = 50)**	**(n = 50)**	**(n = 50)**	
	**Mean (SD)**	**Mean (SD)**	**Mean (SD)**	**Mean (SD)**	**Mean (SD)**	**Mean (SD)**	
12°	9.97 ± 4.10^A^	6.74 ± 2.05^B^	12.27 ± 2.56^C^	9.14 ± 1.21^A^	2.90 ± 1.00^D^	5.07 ± 2.41^E^	0.000*
24°	26.22 ± 4.66^A^	20.10 ± 2.06^B^	30.42 ± 2.49^C^	27.57 ± 1.83^A^	6.19 ± 2.13^D^	26.02 ± 2.34^A^	0.000*
36°	45.14 ± 3.94^A^	39.45 ± 1.99^B^	49.11 ± 2.56^C^	46.96 ± 2.31^D^	16.41 ± 1.73^E^	46.87 ± 1.96^D^	0.000*
48°	71.99 ± 3.89^A^	66.63 ± 1.97^B^	75.38 ± 3.02^C^	73.86 ± 2.62^C^	40.99 ± 2.48^D^	74.49 ± 2.01^C^	0.000*

* Statistically significant for *p* < 0.05. (mean expressed in Nmm). Different letters represent statistically significant differences.

When evaluated at 24°, Bioquick showed the lowest moment (6.19 Nmm), followed by Roth SLI (20.10 Nmm). Shortly after, Roth Max, In-Ovation R and Portia were statistically similar, with values of 26.22, 27.57 and 26.02 Nmm, respectively. Damon 3MX presented the highest moment, 30.42 Nmm (Table 1).

At 36°, Bioquick presented the lowest force *x* deformation ratio (16.41 Nmm), followed by Roth SLI, 39.45 Nmm. Subsequently, Roth Max presented 45.14 Nmm. In-Ovation R and Portia came afterwards, showing a statistically similar behavior (46.96 and 46.87 Nmm, respectively). Damon 3MX showed the highest value (49.11 Nmm) (Table 1).

At 48°, Bioquick confirmed the lowest force *x* deformation ratio (40.99 Nmm), followed by Roth SLI (66.63 Nmm) and Roth Max (71.99 Nmm). In-Ovation R, Portia and Damon 3MX brackets had statistically similar results for this angulation (74.49, 73.86 and 75.38 Nmm, respectively) (Table 1).

### Regression by the least-squares method 

It was observed that torque began to be expressed primarily in Bioquick (1.79°), followed by Roth SLI (3.65°), Roth Max (5.23°), Damon 3MX (5.41°), In-Ovation R (6.91°) and Portia (9.46°). When the values of torque moments were evaluated, it was observed that the effectiveness of torque behaved differently for each bracket type. It was found that, for clinical effects, torque of 5 Nmm first appeared in Damon 3MX, followed by Roth Max, In-Ovation R, Roth SLI, Portia and Bioquick. For a torque of 20 Nmm, the first to be expressed was Damon 3MX, followed by In-Ovation R, Roth Max, Portia, Roth SLI and Bioquick (Tables 2 and 3). 

**Table 2:** - Angle at which the torque expression begins.

**Angle at which torque expression begins**	**Roots of equations of brands (Tartaglia-Cardano's method)**			
**Bracket tested**	**Angle**	**Real root**	**Complex root**	**Conjugated root**
Bioquick	1.79°	1.79	18.75 + 18.90 i	18.75 - 18.90 i
Roth SLI	3.65°	3.65	21.82 + 53.96 i	21.82 - 53.96 i
Roth Max	5.23°	5.23	24.68 + 52.73 i	24.68 - 52.73 i
Damon 3MX	5.41°	5.41	30.86 + 48.10 i	30.86 - 48.10 i
In-Ovation R	6.91°	6.91	31.71 + 50.83 i	31.71 - 50.83 i
Portia	9.46°	9.46	29.63 + 50.72 i	29.63 - 50.72 i

"i" = Square Root of -1.

**Table 3:** - Angles for clinically effective torque range.

**Torque range clinically effective (Nmm)**	**Roots of equations of brands for clinically effective torque (Tartaglia-Cardano's Method)**						
	**Damon 3MX**	**Roth Max**	**In-Ovation R**	**Roth SLI**	**Portia**	**Bioquick**	
Minimum	5.00	7.93°	8.52°	9.62°	10.02°	11.93°	21.4°
		29.61 + 47.48 i	23.24 + 52.20 i	30.36 + 50.22 i	25.01 + 55.71 i	28.39 + 50.28 i	8.94 + 17.70 i
		29.61 - 47.48 i	23.24 - 52.20 i	30.36 - 50.22 i	25.01 - 55.71 i	28.39 - 50.28 i	8.94 - 17.70 i
Maximum	20.00	16.79°	19.48°	19.06°	23.71°	20.19°	39.01°
		25.18 + 46.06 i	17.56 + 51.53 i	25.64 + 48.92 i	31.85 + 61.03 i	24.26 + 49.43 i	0.14 +27.66 i
		25.18 - 46.06 i	17.56 - 51.53 i	25.64 - 48.92 i	31.85 - 61.03 i	24.26 - 49.43i	0.14 - 27.66 i

"i" = Square Root of -1.

The torsions performed at the orthodontic wire, for most brands tested, should range from approximately 8° to 24°, so that effective clinical results can be achieved. Bioquick presented a very distinct behavior from the other brands because even though it began to manifest torque earlier, in order to have moment clinically feasible, it would be necessary to apply greater torsions to the wire used, ranging from 21.4° to 39.01° (Tables 2 and 3).

## DISCUSSION

Torque movement is a key element to obtain good results in orthodontic treatments.^14^ Great concern about its accuracy is directly related to the desired results of occlusion and esthetics for orthodontic treatment. 

In order not to influence the final result, the original torques embedded in the brackets were set to zero through a bonding device that annulled the prescription torque of each bracket. The importance of this procedure has already been reported in the literature seeking distinct forms of parallelism between the wire used in the trials and the accessory slots.^7^^,^ 15 ^,^ 16

The analyses of the test results showed that Bioquick presented the lowest torque value in all angles tested. Damon 3MX presented the highest torque value, up to torsion of 36°, equaling subsequently at 48° with In-Ovation R and Portia (Table 1).

Results showed that the behavior of the brackets tested was not dependent on the type of closure. At 12°, the lowest torque expression was Bioquick's which has an active system, followed by Portia, which is considered to be passive, Roth SLI (active), Roth Max (conventional), In-Ovation R (active), and Damon 3MX (passive), all of which presented the highest torque expression (Table 1). 

This behavior was repeated in subsequent angles, showing no tendency for active, passive or conventional brackets tested, at higher or lower torque expression (Fig 2) (Table 1).

**Figure 3:**
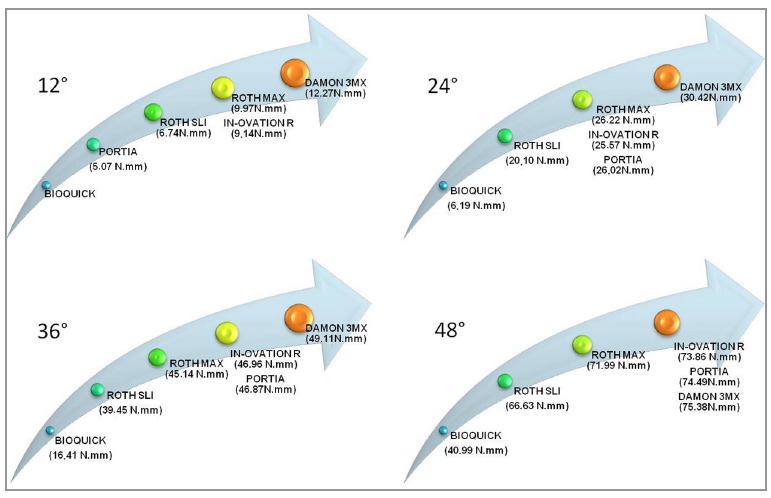
- Intergroup comparison of torque expression.

Regarding active self-ligating brackets, some researches have concluded that they have a higher torque expression than the passive ones.^7^^,^ 17 This happens due to the fact that the clip constantly presses the wire against the bracket slot, especially as the diameter of the arch increases. They also claim that the active self-ligating ones exert a continuous force on the arch, thereby resulting in better accuracy of orthodontic movement and having the ability to reorient themselves three-dimensionally until the arch is completely inserted into the slot.^18^

Some studies, however, corroborated the results found in this research. Morina et al^9^ found only minor differences among the various systems of brackets (active, passive self-ligating and conventional brackets), particularly with regards to their effectiveness in relation to expression and correction of torque.^9^ Pandis et al^11^ and Fansa et al^10^ also found that self-ligating brackets are not superior in relation to conventional ones; in terms of biomechanical characteristics, there are no statistically significant differences between them.^10^

It is important to remember that there are many factors that influence torque during orthodontic treatment: torsion magnitude, wire thickness, slot size, bracket positioning, tooth positioning, wire and bracket composition,^17^ width and depth of the slot, brackets and wires manufacturing tolerance,^5^^,^ 20 difference of constituent leagues of the wires, manufacturing process of brackets (injection-molding, casting, or milling).^20^^,^ 21 All these elements can change the torque expressed in the bracket. Thus, it cannot be said that the active clip, by itself, can effectively increase torque.^16^^,^ 19

The conformation and size of the slot appear to be one of the factors that most influence the effectiveness of torque. In several studies, various measurements indicate that the slots of brackets in general, whether self-ligating or not, are above the size reported by the manufacturer. Therefore, clearance between the wire and the slot can be higher and impair mechanics with regard to the torque expressed.^22^

After regression was performed for each brand separately, and the relevant equations were obtained, the torsion angles in which torque began to be expressed (for a torsion angle equal to zero), as well as in which range of the torsion angle each of these brands could reproduce a torque considered clinically effective, were calculated. It was observed that although torque began to be expressed primarily in Bioquick (1.79°), followed by Roth SLI (3.65°), Roth Max (5.23°), Damon 3MX (5.41°), In-Ovation R (6.91°) and Portia (9.46°), these torques had no capacity to be clinically effective (Tables 2 and 3). 

It is speculated that this early expression of torque depends on slot depth. This means that when the wire comes into contact with the connection system, a torque begins to be expressed earlier, but the wire has not yet found the cervical and incisal walls of the slot. This early generated torque, however, is not able to perform a third-order effective orthodontic movement yet. To achieve clinic effectiveness, the wire must overcome the slot clearance and touch the cervical and incisal walls of the slot.^3^^,^ 23

When the torque moment values of each brand were evaluated over the tests, it was found that the effectiveness of torque behaved differently for each brand. Damon 3MX showed earlier the capacity to express a torque considered clinically effective, i.e., with a 7.93° torsion angle, it could already express 5 Nmm, while Bioquick needs a torsion of 21.4° to achieve the same 5 Nmm. 

Based on the literature, the torque range considered clinically effective indicates moments of force ranging from 5 to 20 Nmm.^3^^,^ 7 It was found that for clinical effects, torque of 5 Nmm first appeared in Damon 3MX, followed by Roth Max, In-Ovation R, Roth SLI, Portia and Bioquick. The first to express a torque of 20 Nmm was Damon 3MX, followed by In-Ovation R, Roth Max, Portia, Roth SLI and Bioquick. This shows that, in order to achieve effective clinical results, the torsions performed on an orthodontic wire, for most brands tested, should range from approximately 8° to 24°. Bioquick presented a very distinct behavior from the other brands evaluated because even though it started to express torque earlier, it would be necessary to apply higher torsions to the wire used, ranging from 21.4° to 39.01°, so that the moment could be clinically feasible (Tables 2 and3).

As there are several factors that influence brackets manufacture, it would be ideal to have a more rigid control in all manufacturing stages, with constant measurements and a more accurate dimensional control. This is because some distortion may occur during the process. Thus, it seems reasonable to assert that the relationship between wire and slot is more important than the ligation systems of different bracket brands regarding effectiveness of torque.^3^^,^ 5 ^,^ 9 ^,^ 22 ^,^ 23 ^,^ 24 It is also worth emphasizing the importance of better understanding the particularities of each bracket in relation to the accuracy of their dimensions, as well as to verify what torque should be given to the wire for each case to be treated, so that a clinically effective torque can be performed in conjunction with the bracket chosen by the orthodontist. 

## CONCLUSIONS

Differences were observed in all torsions studied. Bioquick showed the lowest torque expression in all torsions tested; in contrast, Damon 3MX expressed the highest torque up to 36°. At 48°, In-Ovation R, Portia and Damon 3MX had similar torque moments. 

When the torque range considered clinically effective was observed, it was found that Damon 3MX was the first to express clinically effective torque; in contrast, Bioquick was the last to express a clinically effective torque.

The connection system between wire/bracket (active, passive self-ligating or conventional with elastic ligature) seems not to interfere in final torque expression, the latter being dependent on the interaction between the wire and bracket chosen to be used in orthodontic mechanics.
